# Role of K^+^ and Ca^2+^-Permeable Channels in Osteoblast Functions

**DOI:** 10.3390/ijms221910459

**Published:** 2021-09-28

**Authors:** Hiroaki Kito, Susumu Ohya

**Affiliations:** Department of Pharmacology, Graduate School of Medical Sciences, Nagoya City University, Nagoya 467-8601, Japan

**Keywords:** osteoblast lineage cell, K^+^ channel, Ca^2+^-permeable channel, proliferation, differentiation, osteoblastogenesis

## Abstract

Bone-forming cells or osteoblasts play an important role in bone modeling and remodeling processes. Osteoblast differentiation or osteoblastogenesis is orchestrated by multiple intracellular signaling pathways (e.g., bone morphogenetic proteins (BMP) and Wnt signaling pathways) and is modulated by the extracellular environment (e.g., parathyroid hormone (PTH), vitamin D, transforming growth factor β (TGF-β), and integrins). The regulation of bone homeostasis depends on the proper differentiation and function of osteoblast lineage cells from osteogenic precursors to osteocytes. Intracellular Ca^2+^ signaling relies on the control of numerous processes in osteoblast lineage cells, including cell growth, differentiation, migration, and gene expression. In addition, hyperpolarization via the activation of K^+^ channels indirectly promotes Ca^2+^ signaling in osteoblast lineage cells. An improved understanding of the fundamental physiological and pathophysiological processes in bone homeostasis requires detailed investigations of osteoblast lineage cells. This review summarizes the current knowledge on the functional impacts of K^+^ channels and Ca^2+^-permeable channels, which critically regulate Ca^2+^ signaling in osteoblast lineage cells to maintain bone homeostasis.

## 1. Introduction

Bone modeling initially occurs during skeletal development and comprises two modes: intramembranous ossification and endochondral ossification [1]. During intramembranous ossification, mesenchymal cells are directly converted to osteoblasts, whereas during endochondral ossification, mesenchymal cells are differentiated into cartilage cells before conversion into osteoblasts [1]. Preosteoblasts derived from multipotent mesenchymal osteoprogenitor cells further differentiate into mature osteoblasts according to osteogenic signals. Mature osteoblasts are responsible for bone formation in bone modeling and remodeling. Therefore, the proliferation and differentiation of osteoblast lineage cells play an important role in the regulation of bone homeostasis [2].

Various growth factors and signaling molecules contribute to osteoblastogenesis. For instance, bone morphogenetic proteins (BMP) belonging to the transforming growth factor β (TGF-β) superfamily have a pivotal role in controlling osteoblast differentiation [3]. BMP signaling involves both canonical and non-canonical pathways. Canonical signaling is a SMAD-dependent pathway involving three types of SMADs: receptor-regulated SMADs (R-SMADs), common-mediator SMADs (Co-SMADs), and inhibitory-SMADs. The activation of type I BMP receptors phosphorylates R-SMADs (SMAD1, 5, and 8) to form a heterotrimeric complex with Co-SMAD, SMAD4. In the nucleus, this SMAD complex acts as a transcription factor to regulate the expression of BMP target genes [4]. On the other hand, non-canonical signaling activates SMAD-independent pathways, such as mitogen-activated protein kinase (MAPK) and phosphatidylinositol-3 kinase (PI3K)/Akt [5]. Additionally, integrins, which are heterodimeric transmembrane cell adhesion complexes composed of α and β chains, are expressed in osteoblasts and contribute to bone formation [6]. Integrins promote osteoblast differentiation by interacting with major extracellular matrix components, such as fibronectin and collagen type I (Col1) [7]. The activation of integrin signaling triggers osteogenesis, indicated by the induction of osteoblast genes, such as the transcription factor Runt-related transcription factor 2 (Runx2), phosphorylation of focal adhesion kinase (FAK), and extracellular signal-regulated kinase 1/2 (ERK1/2) [8]. Runx2 forms heterodimers with the core binding factor β (CBFβ) to control the expression of osteoblast marker genes including *Col1a1*, *Spp1*, *Ibsp*, *Bglap2*, and *Fn1*, and regulates multiple steps during osteoblast differentiation [9].

**Figure 1:**
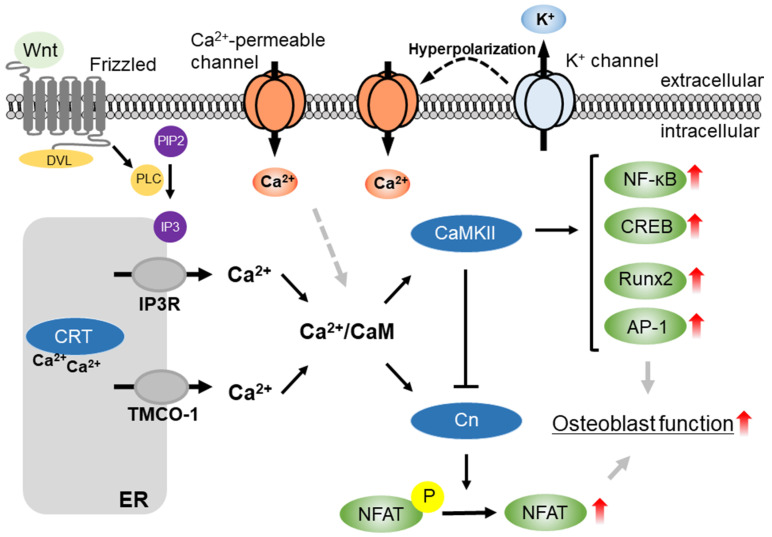
The Wnt/Ca^2+^ signaling is initiated when Wnt ligands bind to the Frizzled receptor. Then, the Disheveled (DVL) is recruited and activates phospholipase C (PLC), leading to Ca^2+^ release. Calreticulin (CRT) is a Ca^2+^-binding multifunctional molecular chaperone in the endoplasmic reticulum (ER). CRT is involved in IP_3_-mediated Ca^2+^ release from ER. [Ca^2+^]_i_ increase via IP_3_R, transmembrane, and coiled-coil domains 1 (TMCO-1), and Ca^2+^-permeable channels cause calmodulin (CaM) activation. The Ca^2+^/CaM complex regulates Ca^2+^/CaM-dependent protein kinase and phosphatase such as CaMKII and calcineurin (Cn). These Ca^2+^-dependent signalings induce the activation of transcriptional factors, including the nuclear factor of activated T cells (NFAT), nuclear factor-kappa B (NF-κB), cAMP response element-binding protein (CREB), Runt-related transcription factor 2 (Runx2), and activation protein 1 (AP-1), leading to the osteoblast differentiation.

Intracellular Ca^2+^ ([Ca^2+^]_i_) signaling has a critical role in osteoblast proliferation and differentiation (Figure 1), mediating direct/indirect Ca^2+^-regulated proteins, such as Ca^2+^/calmodulin-dependent kinase II (CaMKII), a Ca^2+^/CaM-dependent protein phosphatase, calcineurin (Cn), and a Ca^2+^-dependent endoplasmic reticulum (ER) chaperone, calreticulin (CRT). Several studies have shown the significance of Ca^2+^ signaling in osteogenic functions. CaMKII activates cAMP response element-binding protein (CREB)/ activating transcription factor (ATF) and ERK signaling pathways in osteoblasts [10]. Cn controls bone formation and resorption by dephosphorylating the osteogenic transcription regulator, nuclear factor of activated T cells (NFAT) [11,12]. CRT regulates osteoblast differentiation via the NFAT signaling pathway [13]. In addition, the Ca^2+^ leak channel in the ER transmembrane and coiled-coil domains 1 (TMCO1) regulates osteoblast functions via the CaMKII-HDAC4-Runx2 signaling pathway [14]. The non-canonical Wnt signaling pathway is downstream of Ca^2+^ signaling and is involved in intramembranous and endochondral ossification [15]. Wnt-5a activates a non-canonical Wnt pathway by enhancing the activation of Ca^2+^-dependent protein kinase C and Ca^2+^/CaMKII and plays an integral role in BMP2-mediated osteogenic differentiation [16].

In many cases, [Ca^2+^]_i_ mobilization is controlled by intracellular Ca^2+^ store activation and Ca^2+^ influx. Membrane hyperpolarization induced by K^+^ channel activation increases the electromotive driving force of Ca^2+^ entry through voltage-independent, store-operated, Ca^2+^-permeable channels in non-excitable cells [17,18,19]. Therefore, the membrane potential is one of the key biophysical signals in osteoblast lineage cells [20]. The present review summarizes the most recent evidence regarding K^+^ channels and Ca^2+^-permeable channels (Orai/stromal interaction protein (STIM), transient receptor potential (TRP), and mechano-sensing Piezo subfamilies) in osteoblast lineage cells, which are a group of cells comprising mesenchymal progenitors, preosteoblasts, osteoblasts (often called mature osteoblasts), bone-lining cells, and osteocytes, focusing on their role in skeletal development.

## 2. Regulatory Mechanism of Ca^2+^ Signaling by Membrane Potential in Non-Excitable Cell

**Figure 2:**
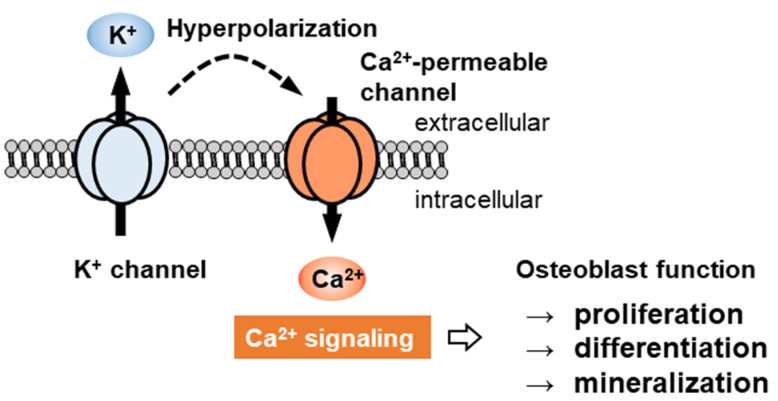
In non-excitable cells, voltage-independent Ca^2+^ channels are the main Ca^2+^-permeable channels. Membrane hyperpolarization followed by K^+^ channel activation increases the driving force for Ca^2+^ influx. Consequently, K^+^ channels indirectly regulate osteoblast functions, including proliferation, differentiation, and mineralization, by controlling intracellular Ca^2+^ signaling in osteoblast lineage cells.

[Ca^2+^]_i_ is a ubiquitous second messenger regulating various cellular functions, such as proliferation, secretion, differentiation, migration, and apoptosis [21]. In quiescent cells, [Ca^2+^]_i_ is maintained at very low levels (50–100 nM). The stimulation of cells with agonists, such as cytokines, hormones, or growth factors, results in [Ca^2+^]_i_ rises via Ca^2+^ influx and/or Ca^2+^ release from the ER and mitochondria. In electrically non-excitable cells, including osteoblast lineage cells, membrane hyperpolarization increases the driving force for Ca^2+^ signaling. A direct link has been demonstrated between membrane hyperpolarization and Ca^2+^ signaling in epithelial cells [22], endothelial cells [23], cancer cells [19], microglia [24], and many types of immune cells [25]. The main Ca^2+^ entry route in these cells is not through voltage-gated Ca^2+^ channels but through Orai or TRP channels with high Ca^2+^ permeability. Membrane hyperpolarization promotes osteogenic differentiation in human mesenchymal stem cells (MSCs) [20], and the K^+^ channels play a pivotal role in the regulation of the membrane potential hyperpolarization. Therefore, a functional analysis of K^+^ channels may provide crucial insights into the functions of osteoblast lineage cells (Figure 2).

## 3. K^+^ Channel Superfamilies in Osteoblast Lineages

**Figure 3:**
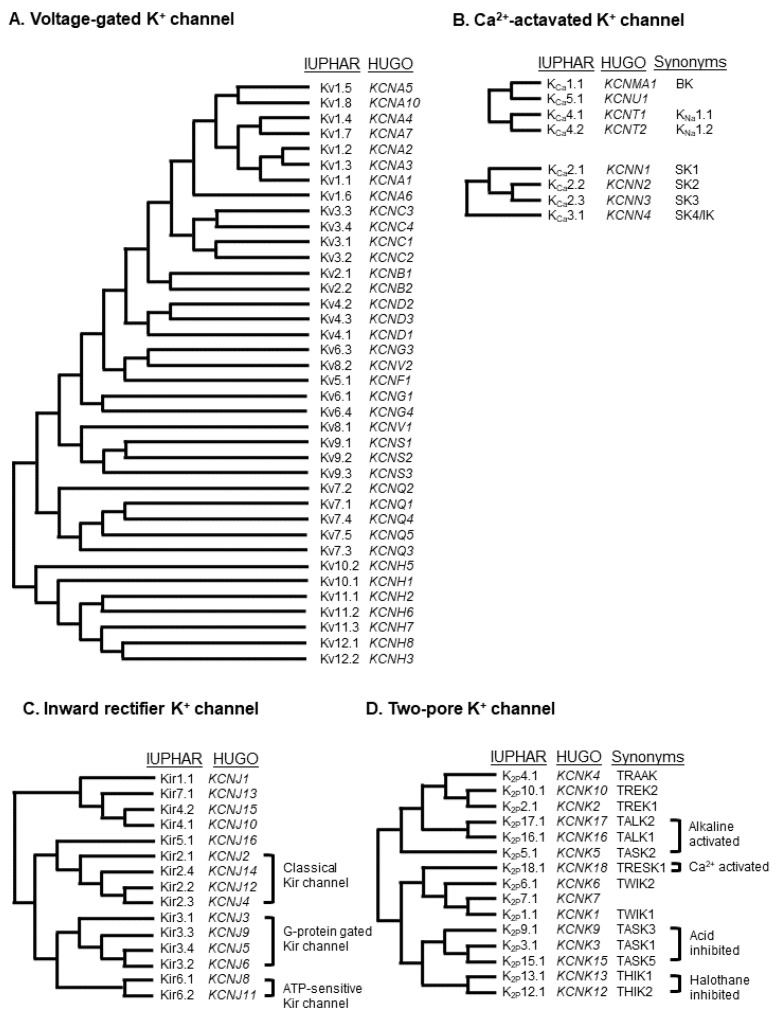
Phylogenetic tree topology of K^+^ channels, voltage-gated K^+^ (K_V_) channels (**A**), Ca^2+^-activated K^+^ channels (**B**), inward-rectifier K^+^ (Kir) channels (**C**), and two-pore K^+^ (K_2P_) channels (**D**).

K^+^ channels are particularly important in maintaining the resting membrane potential and determining the shape and duration of the action potential. K^+^ channels may also regulate the cell volume, proliferation, differentiation, and migration of a wide range of cell types including osteoblast lineage cells (Table 1). Approximately 80 members are classified into voltage-gated K^+^ (K_V_) channel superfamily, inward-rectifier K^+^ (Kir) channel superfamily, Ca^2+^-activated K^+^ (K_Ca_) channel superfamily, and two-pore domain K^+^ (K_2P_) channel superfamily [26] (Figure 3).

K_V_ channel is a tetramer composed of four identical subunits consisting of six transmembrane (TM) (S1–S6) domains with a pore region (S5-P-S6), in which seven amino acidic residues, TTVGYGD that forms the structure of the ion-selectivity filter. K_V_ channels are also characterized by containing a voltage-sensor domain in which the S4 contains positively charged amino acids that constitute the voltage-sensing elements. Kir channel is also a tetramer composed of four identical subunits consisting of two transmembrane domains connected by a pore domain, in which the ion-selectivity filter with the characteristic TXGYG signature sequence. K_Ca_1.1 channels have seven transmembranes (S0–S6). K_Ca_2 and K_Ca_3.1 channels have six transmembranes (S1–S6) like K_V_ channels but also contain an intracellular domain to bind CaM. The pore domain of the K_Ca_1.1 channel is assigned to the region contained between S5 and S6 segments, which include the signature sequence of TVGYG. K_Ca_2 and K_Ca_3.1 channels have six TM segments and a pore loop region (between S5 and S6) containing the characteristic signature sequence GYGD. Then, K_2P_ channel subunits are composed of four TM segments and two pore domains with the characteristic TxGy/FG motif; the 4TM/2P structure defines the membership in the K_2P_ channel family [26].

### 3.1. Voltage-Gated K^+^ (K_V_) Channel Superfamily

The K_V_ channel superfamily is the largest group of K^+^ channel superfamilies. K_V_ channels represent a diverse group of membrane proteins with 12 distinct subfamilies (K_V_1–K_V_12). K_V_ channels are well-known for regulating electrophysiological processes such as action potential generation and excitation-contraction coupling in excitable cells, such as neurons and myocytes [43]. In excitable cells, hyperpolarization followed by K_V_ channel activation reduces [Ca^2+^]_i_ levels by voltage-gated Ca^2+^ channel blockades, thereby inhibiting neurotransmission and muscle contraction. In contrast, in non-excitable cells, hyperpolarization followed by K_V_ channel activation increases [Ca^2+^]_i_ levels by facilitating Ca^2+^ influx through voltage-independent, store-operated Ca^2+^-permeable channels, thereby promoting cell proliferation, migration, and differentiation [43]. K_V_ channels also influence cell cycle progression through cell volume regulation [43].

Yang et al. [27] showed that treatment with tetraethyl ammonium, a K_V_ channel inhibitor, increased the matrix mineralization of human bone marrow-derived mesenchymal stem cells (BMMSCs) during osteogenic differentiation. The pharmacological blockade of K_V_7.3 with linopirdine augmented the mineralization during osteoblast differentiation of osteoblast-like MG-63 and Saos-2 cells through the release of glutamate [27]. Furthermore, several studies have shown that K_V_10.1 (also referred to as *ether-à-go-go*) enhanced the proliferation of MG-63 and Saos-2 cells [28,29]. The functional expression of the K_V_2.1 channel substantially contributed to 17β-estradiol-sensitive K^+^ currents in MG-63 cells [30].

### 3.2. Inward-Rectifier K^+^ (K_ir_) Channel Superfamily

The K_ir_ channel superfamily is best known for its role in maintaining resting membrane potential in cardiac/vascular myocytes, neurons, and pancreatic β cells, regulating muscle contraction/relaxation, action potential firing, and insulin release [44]. K_ir_ channels also regulate bone and cartilage development, as reported by Ozekin et al. [45].

Andersen–Tawil syndrome (ATS) is caused by mutations in the *KCNJ2* gene, which encodes K_ir_2.1, in patients exhibiting cardiac arrhythmia, periodic paralysis, dental defects, cleft lip/palate, micrognathia, hypertelorism, low-set ears, and limb patterning defects. The disruption of K_ir_2.1 also leads to limb defects, hypoplastic craniofacial structures, and cleft palate in mice, craniofacial defects in *Xenopus laevis*, and morphological defects in *Drosophila* [46,47,48]. Recent studies showed that K_ir_2.1 disruption inhibited mammalian facial development by inhibiting BMP signaling [31]. Sacco et al. [32] demonstrated the role of K_ir_2.1 in osteoblast during osteogenesis. Osteoblasts derived from healthy control participants were able to produce bone matrix; however, osteoblasts from ATS patients, as well as in K_ir_2.1 knockout mice, were unable to synthesize bone matrix [32]. On the other hand, the loss of K_ir_2.1 function induced the dephosphorylation of SMAD and inhibited the BMP signaling pathway, resulting in impaired osteoblastogenesis [49].

ATP-sensitive K^+^ (K_ATP_) channels, which are heteromultimers (1:1) of K_ir_6.x, and sulfonylurea receptors, regulate a variety of cellular functions by coupling cell metabolism with membrane potential [44]. Osteogenic differentiation of MSCs strongly upregulated Kir6.2, whereas Kir6.1 and SUR2A showed no significant change. The elevated Kir6.2 expression may contribute to in vitro differentiation of MSC according to the metabolic state of the developing tissue [33]. K_ATP_ channels are related to alkaline phosphatase activity and bone mineralization in rat primary osteoblast cells [34]. The activation of K_ATP_ channels protects osteoblasts from high-glucose-induced injury by promoting osteoblast proliferation and preventing apoptosis [34].

### 3.3. Ca^2+^-Activated K^+^ (K_Ca_) Channel Superfamily

The K_Ca_ channel superfamily consists of eight members: K_Ca_1.1/BK, K_Ca_2.1/SK1, K_Ca_2.2/SK2, K_Ca_2.3/SK3, K_Ca_3.1/SK4/IK, K_Ca_4.1/K_Na_1.1, K_Ca_4.2/K_Na_1.2, and K_Ca_5.1. Due to sequence similarity in the pore region and C-terminus CaM-binding domain, the small-conductance (K_Ca_2.1, K_Ca_2.2, K_Ca_2.3) and intermediate-conductance K_Ca_ (K_Ca_3.1) channels belong to the same genetic lineage (*KCNN* genes) [50]. Distinct from K_Ca_2.x and K_Ca_3.1, the large-conductance K_Ca_ (K_Ca_1.1) has voltage-dependent gating characteristics with sequence similarities to K_V_ channels. K_Ca_1.1 has unique Ca^2+^-binding domains at the C-terminus, RCK1/2 domains (referred to as “Ca^2+^ bowls”), required for Ca^2+^-dependent channel activation [50].

K_Ca_1.1 is activated by depolarization and [Ca^2+^]_i_ elevation. Due to these properties, the K_Ca_1.1 activation results in repolarization and the closing of voltage-dependent Ca^2+^ channels in neurons and smooth muscle cells [51,52]. Therefore, K_Ca_1.1 serves as a negative feedback regulator of membrane potential and [Ca^2+^]_i_ in excitable cells. In contrast, K_Ca_2.2 activation hyperpolarizes and thereby promotes Ca^2+^ influx through TRPC in brain capillary endothelial cells [53]. K_Ca_3.1 activation results in elevated [Ca^2+^]_i_ in human cancer cells [19,54] and immune cells [25]. Taken together, K_Ca_ channels have an important role in the positive feedback mechanism in Ca^2+^ signaling in non-excitable cells.

K_Ca_1.1 especially plays a critical role in osteoclast activation. For instance, K_Ca_1.1 deficiency in mice induced osteopenia due to enhanced osteoclast resorption subsequent to the autonomous release of cathepsin K [55]. K_Ca_1.1 is also functionally expressed in human malignant osteoblast-like osteosarcoma cells (SaM-1, MG-63, and SaOS-2) and osteogenic precursor cells (C1) [35].

Hei et al. [36] showed that K_Ca_1.1-null mice osteoblasts significantly decreased the ability for proliferation and mineralization, thereby decreasing bone mineral density and trabecular bone volume of the tibia and lumbar vertebrae. Integrin-linked kinases regulate bone formation and repair. K_Ca_1.1 proteins interact with integrin β1 in osteoblasts, and the integrin-mediated activation of MAPK-ERK and FAK signaling pathways promotes osteogenesis-related expression of genes, such as Runx2. The blockade of integrin signaling by K_Ca_1.1 knockdown in BMMSCs caused impaired bone formation and osteoblast differentiation [37].

Vascular calcification is a hallmark of cardiovascular disease with chronic kidney disease. One of the key events is the transition of contractile vascular smooth muscle cells (VSMCs) into a non-contractile osteoblast-like phenotype. The activation of K_Ca_1.1 channels ameliorated vascular calcification via Akt/FoxO1 signaling pathways in calcified VSMCs [38]. On the other hand, K_Ca_3.1 channels are expressed in proliferative undifferentiated VSMCs [56]. The pharmacological blockade of K_Ca_3.1 inhibited the calcification medium (Ca^2+^/PO_4_^3−^)-induced upregulation of osterix and osteocalcin in VSMCs and suppressed mineralization during VSMC calcification by simultaneously enhancing osteopontin expression [39] and by interfering with TGF-β and nuclear factor-kappa B (NF-κB) signaling [39].

Many studies have firmly established the important roles of vitamin D (VD) in calcium metabolism and bone development [57]. The VD endocrine system has a beneficial effect on bone homeostasis, but VD receptor (VDR) stimulation has direct positive or negative effects on bone mass depending on the osteoblast development stage. Yamamoto et al. [58] showed that VDR in osteoblasts served as a negative regulator of bone homeostasis due to osteoclastogenesis induced by the receptor activator of NF-κB ligand, RANKL. On the other hand, Sooy et al. [59] reported that osteoblast differentiation was promoted in calvarial osteoblasts obtained from VDR-deficient mice. Our recent study showed that the functional activity of K_Ca_3.1 positively regulated the proliferation of mouse preosteoblast MC3T3-E1 cells by enhancing Ca^2+^ signaling [40]. K_Ca_3.1 activity was suppressed in cells treated with VDR agonists following the downregulation of transcriptional/epigenetic modulators of K_Ca_3.1, such as Fra-1 and HDAC2. Conclusively, a VDR agonist-induced decrease in K_Ca_3.1 activity suppressed cell proliferation in mouse preosteoblasts. Therefore, the combined treatment of K_Ca_3.1 activators and vitamin D preparations may enhance therapeutic effects on bone disorder involving bone mass reduction.

### 3.4. Two-Pore Domain K^+^ (K_2P_) Channel Superfamily

The background leak K_2P_ channel subfamily is involved in diverse physiological functions such as ion homeostasis, hormone secretion, cell development, and excitability and is modulated by mechanical stretch, heat, intracellular/extracellular pH, lipids, and temperature [60]. The human K_2P_ channel subfamily consists of 15 members possessing four transmembrane segments and two-pore-forming domains and assembling as dimers [61]. K_2P_2.1 (tandem of P-domains in a weakly inward rectifying K^+^ (TWIK)-related K^+^ channel 1, TREK1), a mechanosensitive K_2P_ channel member, has been highly expressed in excitable cells [62]. TREK1 is also expressed in human primary osteoblasts and MG-63 cells and is involved in the maintenance of resting membrane potential in human osteoblasts [41]. Of the15 K_2P_ members, five (K_2P_3.1 (TWIK-related acid-sensitive K^+^ channel 1, TASK1), K_2P_5.1 (TASK2), K_2P_9.1 (TASK3), K_2P_16.1 (TWIK-related alkaline pH-activated K^+^ channel 1, TALK1), and K_2P_17.1 (TALK2)) are known to be influenced by extra- and intracellular pH changes [63]. Acid-sensitive K_2P_ channels (K_2P_3.1, K_2P_5.1, and K_2P_9.1), especially, are functionally expressed in MG-63 cells. The extracellular acidosis-induced inhibition of acid-sensitive K_2P_ channel activity reduced MG-63 cell proliferation, indicating that these channels are associated with osteoblast proliferation [42]. The impacts of K_2P_ channels on significant roles in native osteoblast lineages have not been established. However, Cid et al. [64] showed that K_2P_5.1 was expressed in hyaline cartilage from the trachea and the articular surface of the knee. Further studies are expected to provide evidence that K_2P_ channels have physiological and pathophysiological roles in bone/cartilage homeostasis.

## 4. Ca^2+^-Permeable Channel Superfamilies in Osteoblast Lineages

Ca^2+^-permeable channels are present in non-excitable as well as in excitable cells [21] (Table 2). In osteoblast lineages, Ca^2+^-permeable channels play fundamental roles in cellular responses to external stimulation [10,11,12,13,14,15]. A store-operated calcium entry (SOCE), mediated by the activation of Orai/STIM, determines sustained [Ca^2+^]_i_ increase, which is critical in regulating a variety of cellular functions, including proliferation and, more specifically, differentiation of osteoblast lineage cells (Table 3). Mechanical stimuli may also regulate the activation of mechanosensing Ca^2+^-permeable channels such as TRP (Table 4) and Piezo channels (Table 5).

### 4.1. Orai/STIM

Orai1 is a pore-forming subunit of the Ca^2+^ release-activated Ca^2+^ (CRAC) channel that mediates Ca^2+^ influx in most non-excitable cells via a store-operated Ca^2+^ entry (SOCE) mechanism [95]. [Ca^2+^]_i_ changes are important signaling pathways in the regulation of osteoblast linage function [10], but causal roles remain highly controversial.

Studies on Orai1-deficient mice provided the first evidence of the importance of Orai1 for osteoblastic bone formation. The genetic deletion of Orai1 impaired bone development in the mice and resulted in osteopenia due to defective osteoblastic bone formation [65,66]. In addition, Choi et al. [67] showed that Orai1 contributed to the proliferation and differentiation of osteoblast lineage cells at various stages of maturation and that Orai1 deficiency attenuated the proliferation, differentiation, and Col1 secretion of mesenchymal progenitors and primary calvarial osteoblasts in Orai1-deficient mice. Orai1 deficiency also decreased the expression of an osteoid osteocyte marker gene, phosphate-regulating gene with homologies to endopeptidases on the X chromosome (*Phex)*, a mineralizing osteocyte marker gene, dentin matrix protein 1 (*DMP1)*, and a mature osteocyte marker gene, fibroblast growth factor 23 (*FGF*23) in mouse cortical long bone [67]. Taken together, Orai1 plays a crucial role in the differentiation of osteoblast lineage cells from mesenchymal progenitors into osteocytes.

Additionally, Liu et al. [68] showed that overexpression of Orai1 promoted human cartilage-derived MSC differentiation to osteoblasts. Lee et al. [69] reported on the Orai1 contribution to osteogenic effects of BMP2. Differentiation of BMMSCs into bone-forming osteoblasts requires the orchestrated regulation of signaling pathways, such as BMP signaling [96]. BMP signaling induces SMAD1/5/8-dependent signal transduction via BMP receptor types I and II, which activates the expression of Runx2, osterix, and osteoblast differentiation markers [97]. BMP-induced osteogenic conditions failed to induce increased osteogenic differentiation markers in BMMSCs of Orai1-deficient mice. These results suggest that Orai1 regulates osteogenic differentiation through BMP signaling. Interestingly, constitutively activated BMP signaling reversed the inhibition of osteogenic differentiation in Orai1-deficient mice. Collectively, BMP signaling is the downstream pathway of Orai1-mediated osteogenic differentiation.

FGF23, mainly produced by osteocytes and osteoblasts, is a pivotal regulator of renal functions such as phosphate/calcium handling and vitamin D_3_ homeostasis [98,99,100]. SOCE through Orai1 induces FGF23 production in rat osteoblast-like UMR106 cells [70,71]. Glosse et al. [101] showed that the blockade of SOCE decreased upregulated FGF23 expression induced by AMP-dependent kinase (AMPK) inhibition. Therefore, inhibitory AMPK-mediated FGF23 production may be regulated by Orai1-mediated SOCE activity in osteoblasts [101].

Peroxisome proliferator-activated receptors (PPARs) are nuclear transcriptional factors and are composed of PPARα, PPARβ/δ, and PPARγ [102]. PPARα is expressed in many organs, including bone [103]. Stimulation with a PPARα agonist induces AMPK activation in osteoblast lineage cells, resulting in a decrease in SOCE activity, whereas the pharmacological and genetic blockade of PPARα promotes FGF23 expression [104]. Thus, PPARα activation downregulated FGF23 expression through the AMPK-mediated suppression of SOCE in osteoblasts.

The ER Ca^2+^ sensor STIM1 is an essential component of the SOCE process. Overexpression of STIM1 in MC3T3-E1 cells promotes osteoblast differentiation and matrix mineralization with the upregulation of osteogenic markers, such as Runx2, Col1, and BMP4 [72]. Therefore, the loss of STIM1 function leading to SOCE dysregulation may be associated with impaired ER Ca^2+^ signaling in several skeletal- and tooth-related disorders [105]. Furthermore, gain-of-function mutations of Orai1 and STIM1 gave rise to tubular aggregate myopathy (TAM) and Stormorken syndrome (STRMK), forming a clinical spectrum encompassing muscle weakness, myalgia, and cramps, and additional multi-systemic signs, including short stature [106]. Silva-Rojas et al. [107] generated a mouse model carrying the most recurrent STIM1 gain-of-function mutation, STIM1 R304W, found in patients with TAM/STRMK. Moreover, microcomputed tomography analyses revealed a severe skeletal phenotype with thinner and more compact bone in STIM1 R304W mice [107,108]. Increased osteoclastogenesis was observed in bone marrow cells derived from patients with TAM/STRMK [109]. Bone formation and resorption are intricately balanced processes driven by bone-forming osteoblasts and bone-resorbing osteoclasts. Therefore, to gain further in vivo information on the contributions of SOCE activity to osteoblastogenesis, the role of SOCE activity in osteoclastogenesis needs to be comprehensively examined.

### 4.2. TRP Channel Superfamilies

TRP channels are voltage-independent, Ca^2+^-permeable, non-selective cation channels. Approximately 30 members are classified into TRPC (canonical superfamily), TRPV (vanilloid superfamily), TRPM (melastatin superfamily), TRPA (ankyrin superfamily), TRPN (NompC superfamily), TRPML (mucolipin superfamily), and TRPP (polycystin superfamily) [110]. TRP channels are activated by many physical or chemical stimuli (e.g., temperature, membrane potential, and pH). Members of the TRPM, TRPV, and TRPP subfamilies are involved in extracellular calcium homeostasis and intracellular Ca^2+^ signaling in osteoblast lineage cells [111].

#### 4.2.1. TRPM Channels

Shear stress is physiologically generated by blood flow and interstitial fluid [112]. Fluid flow is a potent stimulator of osteoblasts and generally can be of three types: (1) steady (fixed in magnitude and direction); (2) pulsating (oscillating in magnitude but fixed in direction); and (3) oscillatory (oscillating in magnitude and periodically reversing direction). Roy et al. [73] demonstrated that oscillatory fluid flow, but not steady and pulsating flow, induced high Ca^2+^ flicker activity at Ca^2+^ microdomains with a localized high [Ca^2+^]_i_ in MG-63 cells. The localization of TRPM7 in lipid rafts as Ca^2+^ microdomains was critical for Ca^2+^ flicker activity [73]. In human MSCs, TRPM7 stimulated by fluid flow promoted [Ca^2+^]_i_, resulting in osteogenic differentiation [74,75].

Clinical pulsed electromagnetic field stimulation is widely applied to promote bone regeneration in both the United States and Europe as a therapeutic approach for patients with musculoskeletal disorders [113]. Although the underlying mechanisms of electromagnetically induced osteogenesis are not yet completely elucidated, several studies have indicated the benefit of applying electromagnetic stimulation in bone-healing processes. Magnetically induced electrostimulation is an attractive approach to osteogenic differentiation [114] and osteoporosis prevention [115]. Indeed, the stimulation by electric fields upregulated TRPM7 expression, leading to the migration of human osteoblasts from femoral heads of patients undergoing total hip replacement [76].

Mechanical stresses regulate bone formation by upregulating RANKL expression in osteoblasts. Acute mechanical stress induced by hypoosmotic shock promoted TRPM3 and TRPV4 activation, resulting in increased Ca^2+^-mediated RANKL and NFATc1 expressions in primary mouse calvarial osteoblasts [77]. NFATc1 is required for parathyroid hormone (PTH)-related peptide-induced RANKL expression in C2C12 and primary-cultured mouse calvarial cells [116]. The [Ca^2+^]_i_ rise through TRPM3 and TRPV4 as a mechanosensor are critical to NFATc1 activity and the subsequent physiological activity of osteoblasts.

#### 4.2.2. TRPV Channels

TRPV1 deficiency results in the reduced osteogenic differentiation potential of BMMSCs [78]. On the other hand, bone marrow cells derived from TRPV1- and TRPV4-deficient mice showed enhanced osteoblast differentiation activity [79]. Gain-of-function mutations of TRPV4 induced congenital skeletal dysplasias, including metatropic dysplasia (MD) [117]. Patients with MD exhibit short limbs at birth due to a defect in long bone development and progressive kyphoscoliosis. Nonaka et al. [118] reported that the novel gain-of-function mutation TRPV4 L619F was involved in the chondrogenic differentiation of MSCs and accelerated the osteogenic differentiation of MSCs obtained from patients with MD [80]. Osteoblasts expressing TRPV4 L619F exhibited increased [Ca^2+^]_i,_ levels; thus, calcification was enhanced with upregulated Runx2 and osteocalcin expression. Indeed, the expression and nuclear translocation of NFATc1 were upregulated in osteoblasts expressing TRPV4 L619F. These findings indicate that MD-associated disorganized endochondral ossification is induced by disordered osteogenic differentiation through the TRPV4-Ca^2+^/NFATc1 signaling axis [80].

A biologically active N-terminal fragment of human PTH, PTH (1-34), is used clinically to increase bone volume in osteoporosis. Pozo et al. [81] showed that PTH (1-34) promoted Ca^2+^ influx through TRPV4 in MG-63 cells via a PKA-dependent pathway. Interestingly, the PTH-induced Ca^2+^ influx significantly inhibited MG-63 cell migration. When PTH (1-34) is administered to humans or rodents, bone mass was increased due to an increased number of osteoblasts and bone formation rate [119]. PTH (1-34) also suppressed osteoblast apoptosis [120] and converted quiescent bone-lining cells into active osteoblasts [121]. Further studies will be needed to clarify the relationship between cytosolic Ca^2+^ dynamics and osteoblast functions via PTH stimulation.

#### 4.2.3. TRPP Channels

Autosomal dominant polycystic kidney disease is caused by inactivating mutations in the gene that encode either TRPP1 or TRPP2 [122]. TRPP1 binds to TRPP2 through their respective C-terminal coiled-coil domains to form the functional TRPP complex in the cell membrane [123]. This complex between TRPP1 and TRPP2 functions as a mechanosensor in bone and kidney [124].

Recent studies indicated that the TRPP complex played a pivotal role in osteoblastogenesis [82,83,85,86,125]. TRPP1 has been shown as a mechanosensing molecule involved in osteoblastogenesis and bone remodeling. Dalagiorgou et al. [125] reported that mechanical stress upregulated Runx2 expression by activating the TRPP1-JAK2/STAT3 signaling pathway, resulting in osteoblastic differentiation in human osteoblastic cells and, ultimately, bone formation. In addition, TRPP1 and the transcriptional coactivator TAZ formed a mechanosensing complex and contributed to osteoblastic differentiation in MSCs [82,83].

Osteoporosis is a disease characterized by low bone volume, skeletal fragility, and an increased risk of fracture. Bicaudal C homolog 1 (Bicc1) is one of the genetic determinants of patient bone mineral density, and Bicc1-deficient mice had low bone mineral density. Mesner et al. [85] indicated that TRPP2 was a downstream target of Bicc1 involved in osteoblast differentiation. In addition, a human genome-wide association study (GWAS) meta-analysis showed that single nucleotide polymorphisms (SNPs) in TRPP2 are associated with decreased bone mineral density. These findings suggest that Bicc1 is a critical determinant of osteoblastogenesis and bone mineral density by regulating TRPP2 transcription.

The osteoblast-specific depletion of TRPP2 resulted in reduced bone volume associated with decreased bone mineral density, mineral apposition rate, trabecular bone volume, and cortical thickness, and impaired biomechanical properties of bone. TRPP2-deficient osteoblasts exhibited lower basal [Ca^2+^]_i_ and impaired response to flow-induced Ca^2+^ influx [86]. Additionally, the short hairpin RNA (shRNA)-mediated blockade of TRPP1 suppressed Ca^2+^ signaling to shear fluid stress in MG-63 cells [84]. These studies suggest that Ca^2+^-permeable channel TRPP2 is coupled to mechanosensing TRPP1 in response to mechanical stress-loaded osteoblasts during osteoblast differentiation.

### 4.3. Piezo Channels

**Figure 4:**
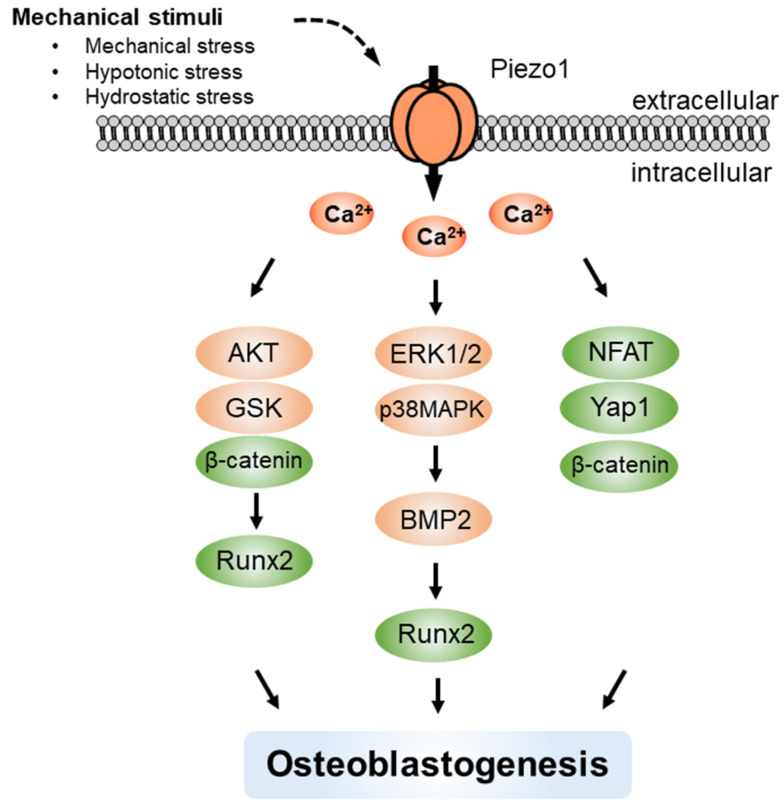
Mechanical stress stimulates Piezo1 to promote Ca^2+^ influx in osteoblast lineage cells. The [Ca^2+^]_i_ rises regulate various signal transduction pathways, resulting in osteoblastogenesis.

Piezo channels are emerging as important regulators of various aspects of mechanosensing [126,127,128]. Piezo2 is predominantly expressed in sensory neurons, such as dorsal root ganglia neurons, whereas Piezo1 is primarily expressed in non-sensory tissues and non-neuronal cells and senses various mechanical stimuli, including shear stress, static pressure, and membrane stretch. Piezo channels are permeable to divalent ions such as Ca^2+^, as well as to monovalent ions [129]. Recent GWAS reports showed that human Piezo1 SNPs were associated with shorter adult height [130] and reduced bone mineral density [131]. Sugimoto et al. [87] showed that hydrostatic pressure induced the differentiation of BMMSCs into osteoblasts with enhanced BMP2 expression via the ERK1/2 and p38 MAPK signaling through Piezo1. In MC3T3-E1 cells, the combined action of both Piezo1 and TRPV4 is involved in the sensing of mechanical fluid shear stress [88], and Piezo1 is essential for Runx2 expression through the Akt/GSK-3β/β-catenin signaling pathway [89]. Indeed, Piezo1 is highly expressed in various bone tissues of mice and plays a critical role in bone formation [90] (Figure 4).

Osteoblast lineage-specific, Piezo1-deficient mice (*Piezo1^OcnCre^*) showed severely impaired bone formation and blunt, mechanical unloading-induced bone loss [90]. Moreover, Piezo1-conditional-deficient mice, *Piezo1^Prrx1Cre^*, exhibited multiple skeletal fractures in long bones, the radius, and ulna; however, Piezo2 conditional-deficient mice (*Piezo2^Prrx1Cre^*) had normal bone development with no fractures. Piezo1/2 double conditional knockout neonatal mice exhibited more severe skeletal defects in the appendicular skeleton [94]. Piezo1 and Piezo1/2 knockout mice showed decreased activity in the Hippo-Yap1/TAZ and Wnt/β-catenin signaling pathway in osteoblasts, thus promoting osteoblastogenic differentiation [94]. In addition, Li et al. [91] reported that osteoblast/osteocyte-specific deletion of Piezo1 decreased bone formation and bone mass in other Piezo1 knockout mice, *Piezo1^Dmp1Cre^*. The shRNA-mediated blockade of Piezo1 in MLO-Y4 osteocyte-like cells reduced the expression of Ptgs2 and Tnfrsf11b [89], well-known targets of fluid shear stress in osteocytes [132,133]. Furthermore, Sasaki et al. [92] reported that mechanical stimulation suppressed an osteocyte-derived negative regulator of bone formation, sclerostin mRNA expression, in murine osteocyte IDG-SW3 cells through the Piezo1-Akt pathway.

Piezo1 knockout mice, *Piezo1^Runx2Cre^*, demonstrated a more severe osteoporotic phenotype than *Piezo1^Dmp1Cre^* [93]. Because Runx2 was expressed in chondrocytes, as well as in osteoblasts, Piezo1 activity in chondrocytes was involved in this aggravation of osteoporosis in *Piezo1^Runx2Cre^* mice [132]. Lee et al. [134] have described the presence of mechanosensory Piezo channels in chondrocytes, which function synergistically in response to injurious mechanical loading.

Wang et al. [135] reported that depletion of osteoblastic Piezo1 promoted bone resorption in *Piezo1^Prx1Cre^* mice. Piezo1 in osteoblasts regulated the YAP-dependent expression of Col types II and IV, which control osteoclast differentiation, suggesting that Piezo1 can coordinate the crosstalk between osteoblasts and osteoclasts by directly sensing mechanical stress in osteoblast lineage cells.

## 5. Conclusions/Future Perspectives

Osteoporosis is a major skeletal disorder that influences bone structure and composition. Gradual bone loss results in bone fragility and significantly increases the risk of bone fracture. An estimated 1.5 million fractures occur per year in the United States [136]. Bone homeostasis depends on intracellular Ca^2+^ signaling, as well as on the external calcium balance that regulates the function and differentiation of chondrocytes, osteoclasts, and osteoblast lineage cells. A number of ion channels critically contribute to the regulation of these processes, including intestinal calcium absorption and renal calcium reabsorption [137].

This review has highlighted the critical role of K^+^ channels and Ca^2+^-permeable channels in osteoblast functions. However, the lack of understanding of their specific functions in bone formation poses a definite limit. Elucidating their involvement in bone signaling pathways could help identify molecules targeting bone biology. Emphasis should be given on data describing the cooperative coupling of K^+^ and Ca^2+^ signaling in regulating the physiological and pathophysiological frameworks of bone homeostasis.

## Figures and Tables

**Table 1:** Functional expression of K^+^ channels in osteoblast lineage cells.

Family	Member	Cell Types	Function	Reference
voltage-gated K^+^ channel	Kv7.3	human mesenchymal stem cells, MG-63, and Saos-2 cells	osteoblast differentiation and mineralization	[27]
	Kv10.1	MG-63 cells	proliferation/ cell cycle progression	[28]
		MG-63, Saos-2, and hFOB 1.19 cells	prolifeation	[29]
	Kv2.1	MG-63 cells	functional expression	[30]
inward rectifier K^+^ channel	Kir2.1	myoblasts form Andersen-Tawil syndrome (ATS) patients	Mineralization	[31]
		induced pluripotent stem cells form ATS patients	osteoblast differentiation and mineralization	[32]
	Kir6.2	human mesenchamal stromal cells	osteogenic diferentiation	[33]
	Kir6/SUR1	rat calvarial osteoblasts	proliferation, apoptosis, and mineralization	[34]
Ca^2+^-activated K^+^ channel	K_Ca_1.1	SaM-1, MG-63, SaOS-2, and HOS cells	functional expression	[35]
		ROS17/2.8 and MC3T3-E1 cells	osteoblast proliferation and differentiation	[36]
		mouse bone marrow mesenchymal stem cells, ROS17/2.8, and MC3T3-E1 cells	bone loss and defects in osteoblast formation	[37]
		rat/mouse vascular smooth muscle cells	vascular calcification (-)	[38]
	K_Ca_3.1	rat/mouse vascular smooth muscle cells	vascular calcification (+)	[39]
		MC3T3-E1 cells	prolifeataion	[40]
two-pore K^+^ channel	K_2P_2.1	human osteoblasts and MG-63 cells	functional expression	[41]
	K_2P_3.1, K_2P_5.1, K_2P_9.1	MG-63 cells	prolifeartion	[42]

**Table 2:** Nomenclature of the Ca^2+^-permeable channel superfamilies.

IUPHAR	HUGO	Synonyms
TRPC		
TRPC1 TRPC2 TRPC3 TRPC4 TRPC5 TRPC6 TRPC7	*TRPC1* pseudogene *TRPC3* *TRPC4* *TRPC5* *TRPC6* *TRPC7*	TRP1 TRP3 TRP4 TRP5 TRP6 TRP7
TRPV		
TRPV1 TRPV2 TRPV3 TRPV4 TRPV5 TRPV6	*TRPV1* *TRPV2* *TRPV3* *TRPV4* *TRPV5* *TRPV6*	VR1 VRL/VRL1 VR3 VR2/TRP12/OTRPC4 ECAC1/CAT2/OTRPC3 ECAC2/CAT1/CATL
TRPM		
TRPM1 TRPM2 TRPM3 TRPM4 TRPM5 TRPM6 TRPM7 TRPM8	*TRPM1* *TRPM2* *TRPM3* *TRPM4* *TRPM5* *TRPM6* *TRPM7* *TRPM8*	LTRPC1/MLSN1 LTRPC2/TRPC7 LTRPC3/ MLSN3 LTRPC4 LTRPC5/MTR1 CHAK2/HOMG1 LTRPC7/CHAK1 LTRPC6/TRPP8
TRPA		
TRPA1	*TRPA1*	ANK/TM1
TRPN		
TRPN1	*trpn1 (fish)*	nompC
TRPML		
TRPML1 TRPML2 TRPML3	*MCOLN1* *MCOLN2* *MCOLN3*	mucolipin1 mucolipin2 mucolipin3
TRPP		
TRPP2 TRPP3 TRPP5	*PKD2* *PKD2L1* *PKD2L2*	polycystin 2 polycystin 2L1 polycystin 2L2
Orai		
Orai1 Orai2 Orai3	*ORAI1* *ORAI2* *ORAI3*	CRACM1/TMEM142A TMEM142B TMEM142C
Piezo		
Piezo1 Piezo2	*PIEZO1* *PIEZO2*	

**Table 3:** Functional expression of Orai/STIM in osteoblast lineage cells.

Member	Cell Types	Function	Reference
Orai1	mouse bone marrow mesenchymal stromal cells and MC3T3-E1 cells	osteoblast differentiation and mineralization	[65]
	human osteoprogenitor cells (CC-2538)	osteoblast differentiation and mineralization	[66]
	mouse calvaria osteoblasts and mesenchymal progenitors	osteoblast differentiation and mineralization	[67]
	human cartilage derived mesenchamal stem cells	osteoblast differentiation	[68]
	mouse bone marrow mesenchymal stromal cells	osteoblast differentiation and mineralization	[69]
	UMR106 cells	FGF23 expression	[70,71]
STIM1	MC3T3-E1 cells	osteoblast differentiation and mineralization	[72]

**Table 4:** Functional expression of TRP channels in osteoblast lineage cells.

Member	Cell Types	Function	Reference
TRPM7	MG-63 cells human mesenchymal stem cells human osteoblasts human osteoblasts	Ca^2+^ flicker activity osteoblast differentiation osteoblast differentiation migration	[73] [74,75] [75] [76]
TRPM3, TRPV4	mouse calvarial osteoblasts	RANKL and NFATc1 expression	[77]
TRPV1	mouse bone marrow stromal cells	osteoblast differentiation and mineralization	[78]
TRPV1, TRPV4	mouse bone marrow cells	osteoblast differentiation	[79]
TRPV4	mesenchymal stem cells from metatropic dysplasia patients MG-63 cells	osteoblast differentiation osteoblast proliferation and differentiation	[80] [81]
TRPP1	human osteoblastic cells MG-63 cells	osteoblast differentiation osteoblast differentiation and mineralization	[82,83] [84]
TRPP1, TRPP2	mouse calvarial osteoblasts	osteoblast differentiation and mineralization	[85]
TRPP2	mouse calvarial osteoblasts	osteoblast differentiation and mineralization	[86]

**Table 5:** Functional expression of Piezo channels in osteoblast lineage cells.

Member	Cell Types	Function	Reference
Piezo1	human mesenchymal stem cells, UE7T-13 cells, and SDP11 cell	osteoblast differentiation	[87]
	MC3T3-E1 cells	proliferation	[88]
	MC3T3-E1 cells	Runx2 expression	[89]
	MC3T3-E1 cells and mouse calvarial osteoblasts	osteoblast differentiation	[90]
	mouse bone marrow stromal cells	osteoblast differentiation	[91]
	MLO-Y4 cells	mechanotransduction	[91]
	IDG-SW3 cells	Sost expression	[92]
	mouse calvarial osteoblasts	osteoblast differentiation	[93]
Piezo1, Piezo2	mouse bone marrow stromal cells	osteoblast differentiation	[94]

## Data Availability

Not applicable.
